# Risks of postoperative respiratory failure in elderly patients after hip surgery: a retrospective study

**DOI:** 10.1186/s13018-022-02909-9

**Published:** 2022-03-04

**Authors:** Jia Chen, Zhi Tian, Huaxing Zhang, Lifang Shi, Wenjuan Bao, Teng Huang, Jinshuai Zhai, Nan Gao, Wenyi Li

**Affiliations:** 1 Department of Orthopedics, Hebei General Hospital, No. 348 Heping East Road, Shijiazhuang, 050051 Hebei China; 2 Department of Nephrology, Hebei General Hospital, Shijiazhuang, Hebei China

**Keywords:** Elderly, Hip fracture, Postoperative respiratory failure, Retrospect, Risk factors

## Abstract

**Background:**

The purpose of this retrospective study was to investigate the determinants of postoperative respiratory failure in elderly patients with hip fracture.

**Methods:**

The subjects of this study were 663 elderly patients who had hip fracture and had been treated with total hip arthroplasty at our hospital from January 2014 to May 2020. According to the occurrence of postoperative respiratory failure, 626 patients with no respiratory failure were retrospectively included in the control group, and 37 cases combined with respiratory failure were enrolled in the PRF group. The clinical and surgical data of the two groups were collected and analyzed to evaluate the determinants of respiratory failure by logistic regression analysis.

**Results:**

There were no significant differences in the demographics and baseline variables including age, gender, fracture type and location between the groups (*P* > 0.05). All patients received hip surgery including total hip arthroplasty (THA), hemiarthroplasty (HA) and internal fixation with PFNA (proximal femoral nail anti-rotation). There were no significant differences in operative time and intraoperative blood loss between the groups (*P* > 0.05). However, close associations were found between pulmonary hypertension (univariate analysis: OR = 3.792, 95% CI = 1.421–10.203; multivariate analysis: OR = 1.132, 95% CI = 1.003–1.251), obstructive pulmonary disease (OR = 1.119, 95% CI = 1.009–1.238; multivariate analysis: OR = 13.298, 95% CI = 4.021–43.298), bronchiectasis and emphysema (OR = 4.949, 95% CI = 1.919–9.873; multivariate analysis: OR = 11.231, 95% CI = 187.87), and history of respiratory failure (OR = 6.098, 95% CI = 2.012–12.198; multivariate analysis: OR = 8.389, 95% CI = 2.391–21.982) with postoperative respiratory failure (*P* < 0.05).

**Conclusion:**

Pulmonary hypertension, abnormal lung texture, obstructive pulmonary disease, bronchiectasis, emphysema, history of respiratory failure, and hypoproteinemia may be risk factors for postoperative respiratory failure in elderly patients with hip fracture.

## Background

With an increasing aging population in China, osteoporotic hip fracture remains a crucial health issue because it is related to a high mortality and morbidity, and costs [1]. However, most elderly patients suffering from hip fracture have different levels of comorbidity; meanwhile, traditional conservative treatment requires prolonged recumbency, and is accompanied with multiple adverse events, and healing is difficult to achieve, and even death may ensue. As a result, it is recommended that surgical treatment should be conducted as early as possible.

Previous data showed that the 1-year mortality rate of elderly patients with hip fracture was 8 times that of the same age group without hip fracture, and the mortality rate of males was significantly higher than that of females [2]. Among various postoperative complications in elderly patients with hip fracture, the incidence of wound infection and internal fixation failure caused by fracture surgery is low. As a matter of fact, respiratory complications, including respiratory failure and pneumonia, are most common, and delirium is the second most common, with an incidence of 4.1–14.1% during hospitalization, and is more harmful [3]. Postoperative respiratory failure (PRF) is one of the most common complications in elderly patients with hip fracture and could lead to longer hospital stays, higher expenses and increased mortality [4].

Considering the high mortality rate of PRF in elderly patients with hip fracture, it has become a key topic of clinical research to identify the risk factors of PRF. However, there has been a paucity of reports on PRF in elderly patients after hip surgery. In order to optimize postoperative outcomes and prevent complications, it is critical to identify patients at high risk of PRF preoperatively. Therefore, this prospective study aimed to explore the determinants of PRF in elderly patients after THA.

## Methods

### Study population

The study protocol was approved by the Institutional Ethics Committee of Hebei General Hospital. The participants were informed about the study and signed written informed consent. All the experimental protocols were conducted in accordance with the Declaration of Helsinki.

The data were collected as part of routine patient care between January 2014 and May 2020. The inclusion criteria were as follows: (1) diagnosis of trochanteric fracture or femoral neck fractures by X-ray, CT, and 3D reconstruction; (2) hip surgery including total hip arthroplasty, hemiarthroplasty and internal fixation with PFNA; (3) ability to stand and walk before fracture; (4) complete clinical data; (5) unilateral hip fracture; (6) hospitalization for surgical treatment; (7) no pathological fracture. The exclusion criteria were as follows: (1) severe coagulation dysfunction; (2) severe femoral proximal deformity; (3) severe hepatic renal failure; (4) complications or concomitant multiple injuries in other organs; (5) cerebrovascular disease within 3 months; (6) combined neurotropic joint disease or autoimmune disease; (7) malignancy; (8) loss to follow-up. Finally, 663 patients were enrolled in this study. The recruited cases were divided into two groups as the control group (*n* = 626) and the PRF group (*n* = 37) according to the occurrence of PRF.

### Observation indicator

General information included age, sex, blood routine and biochemical examination, preoperative bed time, hypertension, cerebrovascular disease, diabetes and renal insufficiency, and pulmonary disease.

Operation-related indicators included operative time and intraoperative blood loss.

### Statistical analysis

Excel 2016 was used to collect data, and SPSS 23.0 was used to analyze the data. Mean ± SD was used to represent measurement data, and t-test was used for analysis. Categorical data were expressed by rate (%), and chi-square test was used for analysis. The risk factors of PRF in elderly patients with hip fracture were analyzed by logistic regression. *P* < 0.05 indicated significant difference.

## Results

### Baseline characteristics

The PRF group included 21 males and 16 females with a range of age of 67–83 years and an average age of (73.03 ± 9.02) years. The fracture types included 11 subtrochanteric fractures, 12 trochanteric fractures and 14 neck fractures. In the control group, 359 males and 267 females aged from 67 to 82 years, with an average age of (73.29 ± 9.09) years. Fracture types included 296 trochanteric fracture and 330 femoral neck fracture. In addition, the types of fracture and position were compared between the two groups. The results showed no difference in these variables. Therefore, the general data of the two groups were comparable (*P* > 0.05, Table 1).

**Table 1:** The baseline characteristics

	PRF group (*n* = 37)	Control group (*n* = 626)	*χ*^2^/*t*	*P*
Age (year)	73.03 ± 9.02	73.29 ± 9.09	− 0.169	0.866
Gender (male/female)	21/16	359/267	0.005	0.944
Fracture type				
Femoral trochanteric fracture	23	296	0.006	0.997
Femoral neck fracture	14	330		
Position				
Left	20	355	0.100	0.752
Right	17	271		

### The comparison of operation-related indicators

There were no significant differences in operative time and intraoperative blood loss between the two groups (*P* > 0.05), as shown in Table 2.

**Table 2:** The comparison of operation-related indicators

	PRF group (*n* = 37)	Control group (*n* = 626)	*t*	*P*
Operation duration (min)	82.91 ± 10.69	82.01 ± 10.58	0.503	0.615
Intraoperative blood loss (mL)	198.73 ± 20.38	196.32 ± 19.83	0.717	0.474

### The comparison of underlying diseases

Comparison of the rates of pulmonary hypertension, hypoproteinemia, obstructive pulmonary disease, bronchiectasis, emphysema and previous respiratory failure history in the two groups showed significant differences (*P* < 0.05). However, no difference was found in the rates of hypertension and diabetes, and preoperative bed time between the two groups (*P* > 0.05). The comparisons of underlying diseases are shown in Table 3.

**Table3:** The comparison of underlying diseases

	PRF group (*n* = 37)	Control group (*n* = 626)	t	*P*
Pulmonary hypertension (*n*)	11	48	20.974	< 0.001
Obstructive pulmonary disease (*n*)	5	1	69.467	< 0.001
Bronchiectasis and emphysema (*n*)	8	2	106.714	< 0.001
Previous history of respiratory failure (*n*)	7	2	90.254	< 0.001
Hypertension (*n*)	5	107	0.319	0.572
Diabetes (*n*)	9	151	0.001	0.978
Bed time (*d*)	6.92 ± 2.31	6.89 ± 2.42	0.073	0.942
Hypoproteinemia (*n*)	16	126	11.091	0.001

### The potential risk factors of PRF in regression analysis

Taking the occurrence of PRF as the dependent variable and the factors with differences in the above data, such as pulmonary hypertension, hypoproteinemia, obstructive pulmonary disease, bronchiectasis and emphysema, and previous history of respiratory failure as independent variables, univariate logistic regression analysis showed that pulmonary hypertension, obstructive pulmonary disease, bronchiectasis and emphysema, and history of respiratory failure were all risk factors of PRF in elderly patients after hip surgery, and the differences were significant (*P* < 0.05), as shown in Table 4. Multivariate logistic regression analysis showed that pulmonary hypertension, obstructive pulmonary disease, bronchiectasis and emphysema, and previous history of respiratory failure were all risk factors of PRF in elderly patients with hip fracture, with significant differences (*P* < 0.05), as shown in Table 5.

**Table 4:** The potential risk factors of PRF in univariate analysis

	Wald *χ*^2^	*P*	OR (95% CI)
Pulmonary hypertension	6.059	0.012	3.792 (1.421–10.203)
Hypoproteinemia	7.819	0.103	0.765 (0.212–1.231)
Obstructive pulmonary disease	4.782	0.024	1.119 (1.009–1.238)
Bronchiectasis and emphysema	11.029	0.001	4.949 (1.919–9.873)
Previous history of respiratory failure	9.783	0.002	6.098 (2.012–12.198)

**Table 5:** The potential risk factors of PRF in multivariate analysis

	Wald *χ*^2^	*P*	OR (95% CI)
Pulmonary hypertension	4.081	0.039	1.132 (1.003–1.251)
Obstructive pulmonary disease	15.276	< 0.001	13.298 (4.021–43.298)
Bronchiectasis and emphysema	25.672	< 0.001	45.387 (11.231–187.87)
Previous history of respiratory failure	11.029	0.001	8.389 (2.391–21.982)

## Discussion

In this study, the proportion of elderly patients with hip fracture complicated with PRF was 5.58% (37/663), which is consistent with the results of Hung’s research [3]. Due to the characteristics of elderly patients with hip fracture, various complications easily develop. Hip surgery is one of the effective methods for the treatment of hip fracture, which can effectively relieve pain, improve joint function, and enhance the patients’ quality of life [5]. Respiratory failure is a common complication in elderly patients with hip fracture, which not only affects the normal physical function and metabolism of patient, but also has an important influence on the prognosis of elderly patients with hip fracture.

However, the mechanism of PRF after hip surgery remains unclear and may be the result of multiple factors. At present, there are few clinical studies on PRF in elderly patients with hip fracture. To our knowledge, this is the first study to analyze the risk factors of PRF divided into groups after hip surgery in a large sample population. According to an review, alterations in respiratory physiology associated with aging must be appreciated to anticipate and minimize potential complications associated with surgery and anesthesia in the elderly [6].

In our study, all elderly patients received intraspinal anesthesia to minimize intraoperative respiratory perturbations. However, older patients are accompanied by underlying diseases that cannot be avoided, like hypertension, diabetes, malnutrition, and cardiopulmonary disease. Meanwhile, the results of this study showed that the rates of pulmonary hypertension, obstructive pulmonary disease, bronchiectasis and emphysema, history of respiratory failure and hypoproteinemia were significant different between the control group and the PRF group (*P* < 0.05). It is suggested that PRF is more likely to occur in elderly patients with pulmonary hypertension, hypoproteinemia, obstructive pulmonary disease, bronchiectasis and emphysema, history of respiratory failure, and hypoproteinemia after hip surgery.

Pulmonary hypertension is a common disease of the body's respiratory and circulatory system. Increased pulmonary vascular pressure and resistance, and even vessel occlusion and muscular pulmonary vascular plexus lesions not only restrict the body's respiratory function, but also induce respiratory exhaustion and death [7].

In addition, especially elderly patients, due to the deterioration of physical function, face the phenomenon of cardiopulmonary function compensation, reduced stress ability and anti-hypoxia ability, further aggravating disease progression. Hypoproteinemia is one of the important indicators affecting acute diseases. Due to the poor viscera function in elderly patients and the need for anesthesia during hip surgery, liver function is further affected and albumin synthesis is inhibited. In addition, because surgery increases capillary permeability, resulting in increased albumin leakage. Moreover, catabolism increases and albumin consumption rises under surgical stress [8]. Data showed that hypoproteinemia is very common in elderly patients with hip fracture after surgery, and hypoproteinemia in elderly patients is easy to induce serious complications such as heart failure, pleural effusion, lung infection and brain dysfunction, which is not conducive to postoperative recovery [9].

Chronic obstructive pulmonary disease (COPD) is still a common disease globally. Patients have characteristic restricted respiratory air flow, damage to the lung function and irreversible development sustainability. Respiratory failure is the end stage of COPD. Respiratory failure is hard to cure and death easily occurs because of the compled etiology of respiratory failure [10, 11]. Furthermore, one study showed that a history of COPD, dyspnea at rest, dependent functional status, and malnutrition were independently associated with higher rates of PRF [12]. Therefore, it is suggested that respiratory failure is more likely to develop in hip fracture patients with COPD.

Bronchial dilatation is caused by a variety of bronchial diseases and irresupence expansion, and emphyloedema can damage the alveoli and the disease causes the patient to breathe oxygen and discharge carbon dioxide, leading to hypoxia, lung damage, and breathing difficulties, finally respiratory failure, and may even jeopardize the patient's life.

Our univariate logistic regression analysis showed that pulmonary hypertension, obstructive pulmonary disease, bronchiectasis and emphysema, and history of respiratory failure were all risk factors of PRF in elderly patients with hip fracture. Our multivariate logistic regression analysis further revealed that pulmonary hypertension, obstructive pulmonary disease, bronchiectasis and pulmonary emphysema, a history of respiratory failure and hypoalbuminemia were independent risks of elderly patients. It is suggested that the risk factors of PRF in elderly patients with hip fracture include pulmonary hypertension, obstructive pulmonary disease, bronchiectasis and emphysema, and history of respiratory failure.

This study further analyzed surgical indicators and found no difference in operative time, incision length, intraoperative blood loss, postoperative drainage volume and time to ambulation between the two groups. It is suggested that PRF in elderly patients with hip fracture may not be related to surgical indicators, but the specific mechanism needs further study.

However, due to the small number of patients involved and single-center study, there may be group bias, and some indicators cannot fully reflect the overall situation of hip fracture, which should be confirmed by a randomized controlled multi-center study in the future.

## Conclusion

The risk factors of PRF in elderly patients after hip surgery included pulmonary hypertension, obstructive pulmonary disease, bronchiectasis and emphysema, and a history of respiratory failure. Therefore, surgeons should pay attention to the above factors in elderly patients. A detailed and comprehensive preoperative evaluation is essential. As for the adjustable indicators, preoperative adjustment of the patient's physiological status to acceptable range, consultation with relevant departments, and effective non-surgical treatment will improve the quality of life of patients as much as possible.

## Data Availability

The datasets used and/or analyzed during the current study are available from the corresponding author on reasonable request.

## Abbreviations

ARDS: Acute respiratory distress syndrome
PRF: Postoperative respiratory failure
THA: Total hip arthroplasty
