# Correction to: Risks of postoperative respiratory failure in elderly patients after hip surgery: a retrospective study

**DOI:** 10.1186/s13018-022-03164-8

**Published:** 2022-05-25

**Authors:** Jia Chen, Zhi Tian, Huaxing Zhang, Lifang Shi, Wenjuan Bao, Teng Huang, Jinshuai Zhai, Nan Gao, Wenyi Li

**Affiliations:** 1 Department of Orthopedics, Hebei General Hospital, No. 348 Heping East Road, Shijiazhuang, 050051 Hebei China; 2 Department of Nephrology, Hebei General Hospital, Shijiazhuang, Hebei China

## Correction to: Journal of Orthopaedic Surgery and Research (2022) 17:140 10.1186/s13018-022-02909-9

Following publication of the original article [1], an error was identified in the Methods and Results section.

The updated Methods and Results are given below and the changes have been highlighted in **bold typeface**.

Methods: The subjects of this study were 663 elderly patients who had hip fracture and had been treated with **hip surgery** at our hospital from January 2014 to May 2020. According to the occurrence of postoperative respiratory failure, 626 patients with no respiratory failure were retrospectively included in the control group, and 37 cases combined with respiratory failure were enrolled in the PRF group. The clinical and surgical data of the two groups were collected and analyzed to evaluate the determinants of respiratory failure by logistic regression analysis.

Results

Baseline characteristics

The PRF group included 21 males and 16 females with a range of age of 67–83 years and an average age of (73.03 ± 9.02) years. The fracture types included **23** trochanteric fractures and 14 neck fractures. In the control group, 359 males and 267 females aged from 67 to 82 years, with an average age of (73.29 ± 9.09) years. Fracture types included 296 trochanteric fracture and 330 femoral neck fracture. In addition, the types of fracture and position were compared between the two groups. The results showed no difference in these variables. Therefore, the general data of the two groups were comparable (P > 0.05, Table 1). The original article has been revised.

